# Closed-loop mechanical ventilation for lung injury: a novel physiological-feedback mode following the principles of the open lung concept

**DOI:** 10.1007/s10877-017-0040-0

**Published:** 2017-06-26

**Authors:** David Schwaiberger, Philipp A. Pickerodt, Anake Pomprapa, Onno Tjarks, Felix Kork, Willehad Boemke, Roland C. E. Francis, Steffen Leonhardt, Burkhard Lachmann

**Affiliations:** 1 Department of Anesthesiology and Intensive Care Medicine, Campus Charité Mitte and Campus Virchow-Klinikum, Charité –Universitätsmedizin, corporate member of Freie Universität Berlin, Humboldt Universität zu Berlin, and Berlin Institute of Health, Augustenburger Platz 1, Berlin, 13353 Germany; 2 Philips Chair for Medical Information Technology, Helmholtz-Institute for Biomedical Engineering, RWTH Aachen University, Pauwelsstrasse 20, Aachen, 52074 Germany; 3 Department of Anesthesiology and Institute for Molecular Cardiovascular Research, University Hospital RTWH Aachen, Pauwelsstrasse 30, Aachen, 52074 Germany

**Keywords:** Closed-loop mechanical ventilation, Acute respiratory distress syndrome, Lung protective ventilation, Open lung approach, Electrical impedance tomography

## Abstract

Adherence to low tidal volume (V_T_) ventilation and selected positive end-expiratory pressures are low during mechanical ventilation for treatment of the acute respiratory distress syndrome. Using a pig model of severe lung injury, we tested the feasibility and physiological responses to a novel fully closed-loop mechanical ventilation algorithm based on the “open lung” concept. Lung injury was induced by surfactant washout in pigs (n = 8). Animals were ventilated following the principles of the “open lung approach” (OLA) using a fully closed-loop physiological feedback algorithm for mechanical ventilation. Standard gas exchange, respiratory- and hemodynamic parameters were measured. Electrical impedance tomography was used to quantify regional ventilation distribution during mechanical ventilation. Automatized mechanical ventilation provided strict adherence to low V_T_-ventilation for 6 h in severely lung injured pigs. Using the “open lung” approach, tidal volume delivery required low lung distending pressures, increased recruitment and ventilation of dorsal lung regions and improved arterial blood oxygenation. Physiological feedback closed-loop mechanical ventilation according to the principles of the open lung concept is feasible and provides low tidal volume ventilation without human intervention. Of importance, the “open lung approach”-ventilation improved gas exchange and reduced lung driving pressures by opening atelectasis and shifting of ventilation to dorsal lung regions.

## Introduction

The Acute Respiratory Distress Syndrome (ARDS) Network landmark study proved that application of physiological principles translate into highly significant mortality reduction in patients suffering from ARDS [1]. Reduction of tidal volumes (V_T_) and limitation of inspiratory pressures in conjunction with a fixed combination of positive end-expiratory pressure (PEEP) and fraction of inspired oxygen (F_I_O_2_) were used to limit the volu-, baro- and biotrauma caused by mechanical ventilation [2]. Numerous further therapeutic interventions have failed to improve the outcome of patients with severe ARDS. Of note, a recent secondary analysis of human studies applying higher than traditional PEEP values to maintain injured lungs open and thus prevent cyclic alveolar collapse and reopening, indicates increased survival for patients responding to higher PEEP with increased oxygenation of arterial blood [3]. These findings again underline the importance of maintaining uncollapsed alveoli open in severe ARDS. Yet, controversy exists on how to open up the lung and whether recruitment of previously collapsed alveoli should be achieved through short-term application of high inspiratory pressures in conjunction with PEEP levels sufficient to keep the lung open [4].

Regardless, adherence to low tidal volume ventilation and thus to proven standards of lung protective ventilation is low and impeded by the density of work processes in modern intensive care units (ICU) [5]. Thus, provision of lung protective ventilatory strategies by automatized closed-loop ventilation control may help to assure non-injurious low V_T_ ventilation and to keep the lung open.

The purpose of this study was (a) to integrate a “lung open” ventilatory strategy to fully automatized closed-loop mechanical ventilation and (b) to test the feasibility and physiological responses to this novel mechanical ventilation algorithm in a pig model of severe lung injury [6].

## Materials and methods

### Animal preparation

After approval by the local authorities (Approval-No: G 0151/10; G 0145/12), a total of eight animals were used. After premedication with intramuscular injection of Ketamine (30 mg/kg; Ursotamin, Serumwerk Bernburg AG, Bernburg), Xylazin (4 mg/kg; Bayer Vital GmbH, Leverkusen), Azaperon (120 mg, Stresnil, Janssen-Cilag GmbH, Neuss) and Atropine (0.5 mg; Atropinsulfat, B. Braun Melsungen AG, Melsungen), a venous catheter (B. Braun Vasofix Braunüle, 20G) was inserted via an ear vein. Intravenous (i.v.) anesthesia was induced using Propofol (2 mg/kg i.v.; B. Braun Melsungen AG, Melsungen), Fentanyl (0.01–0.04 mg/kg i.v.; Janssen-Cilag GmbH, Neuss) and maintained with Thiopental (14–20 mg/kg/h i.v.; Trapanal; Inresa Arzneimittel GmbH, Freiburg im Breisgau, Germany), Fentanyl (2–6 µg/kg/h i.v) and Pancuronium (4 mg/h i.v.; Inresa Arzneimittel GmbH, Freiburg im Breisgau, Germany). Three lead electrocardiogram (ECG) and peripheral oxygen saturation (SpO_2_) were constantly recorded (data acquisition: PC PPC-154T, Advantech Co., Ltd, Teipei, Taiwan; measuring device: Sirecust 960; Siemens AG, Munich, Germany). The pigs were placed supine and remained in this position throughout the experiment. During the instrumentation, animals were ventilated via a tight fitting face mask connected to a mechanical ventilator (Evita XL; Dräger Medical Deutschland GmbH, Lübeck, Germany) that allowed the application of F_I_O_2_ 1.0, PEEP 5 cm H_2_O, V_T_ 6 ml/kg, inspiratory to expiratory time ratio (I:E) set to 1:2 and an end-tidal CO_2_ (EtCO_2_; CO_2_SMO+, Philips Respironics, Best, The Netherlands) goal of 35–45 mmHg. After local anesthesia with Lidocain 2%, (B. Braun Melsungen AG, Melsungen, Germany) pigs were tracheotomized (9.0 ID tube; Mallinckrodt™; Covidien Deutschland GmbH, Neustadt, Germany) and the right carotid artery and jugular vein were surgically exposed. After insertion of a catheter in the carotid artery for continuous measurement of intra-arterial oxygen saturation (SaO_2_) (CeVOX; Pulsion Medical Systems SE, Feldkirchen, Germany) SaO_2_ and arterial blood pressure were continuously recorded. Next, a central venous catheter was inserted into the external jugular vein for continuous administration of anesthesia and central venous pressure (CVP) measurements. An electronic calibration of all measured vascular pressures was performed after positioning of the pressure transducers (Xtrans^®^; CODAN Critical Care GmbH, Lensahn, Germany) at the mid-thoracic level. The urinary bladder was catheterized with a self-retaining Foley catheter. A 30 min hemodynamic stabilization period was begun after instrumentation of the animals.

### Lung injury model

At baseline, animals were ventilated with a PEEP of 5 cm H_2_O, a V_T_ of 6 ml/kgBW, and EtCO_2_ of 35–45 mmHg. Baseline measurements of hemodynamic, respiratory and standard gas exchange parameters were performed at F_I_O_2_ of 1.0. The experimental protocol started when the ratio of arterial partial pressure of oxygen (PaO_2_) to fraction of inspired oxygen (PaO_2_/F_I_O_2_) was >450 mmHg and the PaCO_2_ was 40–45 mmHg. Basal fluid administration was performed using continuous infusion of 10 ml/kg/h of ringer’s acetate. In addition, norepinephrine was used after lung injury induction to maintain a mean arterial blood pressure (MAP) of 80–85 mmHg. Arterial blood gases were measured in 30 min intervals (ABL 700, Radiometer, Copenhagen, Denmark). For analysis of acid-base balance, the hydrogen ion concentrations corresponding to each individual arterial pH value were averaged. Subsequently, the resulting mean H^+^ concentrations were converted into pH. Standard respiratory parameters were recorded continuously.

After 30 min of baseline ventilation, the lungs were lavaged using heated 0.9% saline solution (40–50 ml/kg, 38 °C). Lavages were repeated until the PaO_2_/F_I_O_2_-ratio was <100 mmHg. The automatized closed-loop ventilation protocol was started when the PaO_2_/F_I_O_2_-ratio remained below 100 at F_I_O_2_ of 1 and PEEP = 5 cm H_2_O for 15 min after the last lavage. All animals were assigned to a fully automatized closed-loop feedback protocol according to the “open lung concept” of Lachmann et al. [4, 7]. The total length of the experimental protocol was 6 h and all animals were euthanized by an overdose of intravenous thiopental, fentanyl and potassium chloride at the end of the experiment.

### System setup and communication protocol

**Fig. 1:**
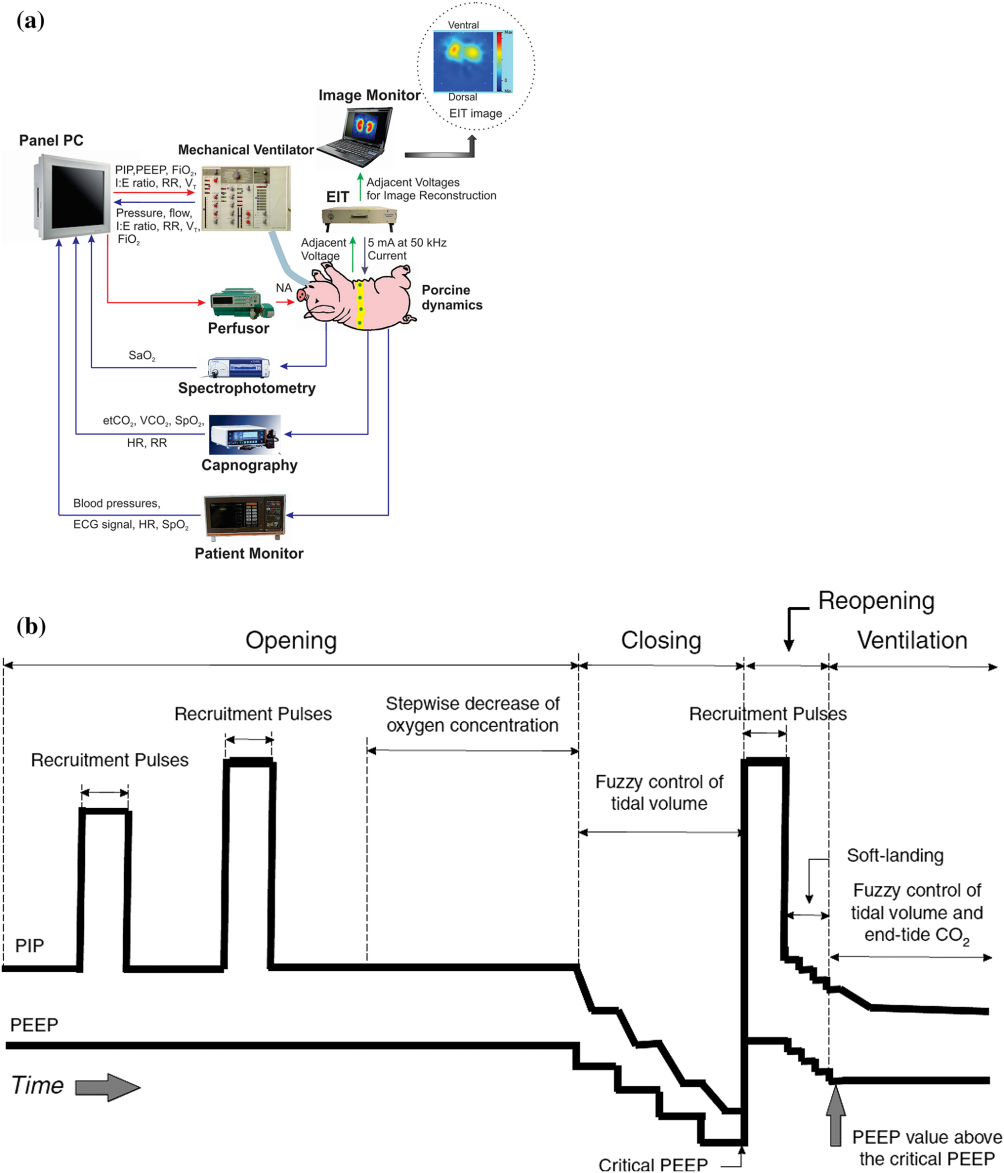
**a** System configuration and data flow for the automatized closed-loop control of mechanical ventilation. *Blue arrows* indicate signal flow to the controller (afferent signals) and *red arrows* indicate signal flow from the controller to steer the mechanical ventilation and norepinephrine infusion (efferent signals). Adapted and reproduced with permission from [8]. **b** Closed-loop mechanical ventilation using the open lung approach (OLA). A brief opening phase is used to recruit previously collapsed alveoli and is followed by identification of the critical PEEP value below which lung derecruitment occurs (closing phase). Lung recruitment is repeated (reopening) with the PEEP value set 2 cm H_2_O above the closing PEEP, followed by mechanical ventilation controlled by closed-loop physiological feedback algorithms (ventilation phase). *PIP* peak inspiratory pressure, *PEEP* positive end-expiratory pressure

The fully automated closed-loop physiological feedback system is characterized by a star topology, where all actuators and all measuring devices are connected to a personal computer (PC) (PPC-154T, Advantech Co., Ltd., Taiwan). The respirator (Servo 300, Maquet Critical Care AB, Sweden) is controlled by this PC as previously described [6, 7]. The PC reacts protocol-driven to any measured input change in respiratory (tidal volume, airway pressures) and oxygenation (SpO_2_, SaO_2_, EtCO_2_) parameters and adjusts the V_T_, PEEP, respiratory rate (RR), I:E-ratio and F_I_O_2_ accordingly. System input to the PC is derived from a capnography device with pulse oximetry (CO_2_SMO+, Novametrix Medical Systems Inc., USA), a monitor for measurements of ECG, systemic arterial blood pressure, SpO_2_ (Sirecust 960, Siemens AG, Germany) and a spectrophotometry device for continuous measurement of intra-arterial oxyhemoglobin saturation (SaO_2_) measurement (CeVOX, Pulsion Medical Systems SE, Germany). These inputs are completed by electrical impedance tomography (EIT) measurements obtained from an EIT device. All data communication protocols (except for the EIT connection) are based on an asynchronous RS232 interface with a sampling time of 100 ms, whereas the EIT system physically connects to the PC by a RJ45 connector using a TCP/IP protocol. The protocols are coded by graphical programming with LabVIEW 7.1 software (National instruments, USA) and fuzzyTECH 5.4 software (INFORM GmbH, Germany). Figure 1a depicts the system set-up and the data flow within the closed-loop physiological feedback system.

### Closed-loop mechanical ventilation: open lung approach protocol (OLA group)

Using the OLA protocol, all tidal volumes are delivered by a decelerating gas flow (pressure-controlled mode). As implemented, the OLA protocol consists of four parts and is based on the “open up the lung and keep it open” concept by Lachmann [4]. First, a titration algorithm is used to search for an appropriate peak inspiratory pressure (PIP) to open recruitable areas of the lung (opening phase). During this opening phase, the PEEP is set to 20 cm H_2_O before the recruitment procedure and a series of recruitment pressures is applied starting by setting the PIP to 45 cm H_2_O for 3–5 breaths. Next, the SaO_2_ is measured after 30 s, while the tidal volume is reduced to 6 ml/kg BW with the PEEP maintained at 20 cm H_2_O. Only if the measured SaO_2_ does not reach a value above 95%, the first recruitment pulse is followed by a second impulse using a PIP of 50 cm H_2_O. If necessary, this stepwise increase of recruitment pressures is applied in steps of 5 cm H_2_O until a maximum pressure of 70 cm H_2_O is reached. Next, the critical PEEP below which lung derecruitment occurs is identified by titrating the PEEP downwards (from 20 cm H_2_O) in steps of 1 cm H_2_O (30 s intervals) until the measured SaO_2_ falls below 90%. The PEEP value corresponding to this arterial saturation measurement is used as the critical derecruitment PEEP of the lung. Once the critical closing PEEP is identified, a reopening phase is used to reopen the lung (reopening phase) by applying the previously identified opening pressures and a PEEP value 2 cm H_2_O above the critical closing PEEP value. All steps of the OLA protocol used to open up- and keep the lung open are performed fully automatically and equally to all animals. Next, a steady state ventilation phase is begun with I:E ratio fixed at 1:1 and the V_T_ and EtCO_2_ regulated to 6 ml V_T_BW and 35 mmHg respectively (ventilation phase). A so called “fuzzy” control of the system is used to achieve both, adjustment of V_T_ by appropriate PIP application and selection of RR based on continuous measurement of EtCO_2_. Using a fuzzy control of the closed-loop system, it is not necessary to establish a precise mathematical model of how to achieve the target values (PIP, PEEP, V_T_, EtCO_2_). For a more detailed description of the deterministic finite automaton and fuzzy control of this closed-loop algorithm, a technical demonstration has previously been published based on the data of one animal [7]. Figure 1b depicts the closed loop OLA protocol.

### Electrical impedance tomography (EIT)

The EIT system (Goe-MF II, Dräger AG, Lübeck, Germany) consists of an acquisition unit, a silicone belt of 16 skin electrodes for current injections and voltage measurements and a host personal computer. The system is attached to the animal via a belt of equidistant electrodes surrounding the chest at the sixth intercostal space. During one cycle of operation, a current of 5 mA with 50 kHz is alternately applied at each adjacent pair of electrodes and thus 16 injections are required per cycle. During each cycle, the adjacent voltages from the remaining electrode pairs are measured. Hence, using a frame rate of 13 frames per second, in one cycle 208 voltage measurements (16 × 13) are used for image reconstruction based on a backprojection algorithm. A series of these EIT images therefore provide the information of electrical impedance within the thorax in real time. EIT pictures were separated in three different regions of interest (ROI) ranging from ventral (ROI 1) to dorsal (ROI 3) regions of the lung.

### Statistical analysis

Due to the small sample size, a normal distribution could not be assumed. Thus, continuous variables are reported as median and interquartile range (IQR) and non-parametric testing was applied: Continuous variables were compared using the Mann–Whitney *U* test and frequencies were compared using Fisher’s exact test. Longitudinal data was analyzed using non-parametric ANOVA methods as described by Brunner et al. to test for interactions of variables with time [9]. Tests were conducted using R 3.1 (http://www.r-project.org). For clarity of the text values are given as mean ± SE. Figures were created using the GraphPad Prism 6.0 software (GraphPad Software Inc., La Jolla, CA, USA).

## Results

### Baseline characteristics

Mean animal body weight was: 27.2 ± 1.4 kg. At the end of baseline ventilation, PaO_2_/F_I_O_2_-ratio was 565 ± 10 mmHg and PaCO_2_ was 45 ± 1 mmHg in healthy pigs. Arterial pH value (calculated from the mean hydrogen ion concentration in arterial blood) at the end of baseline ventilation was 7.481.

During baseline ventilation, tidal volumes were 6.1 ± 0.1 ml/kg BW. At measured PEEP values of 6 ± 0.4 cm H_2_O, the resulting peak inspiratory pressure was 15 ± 0.3 cm H_2_O. Dynamic compliance (C_dyn_) after baseline was 20 ± 1 ml/cm H_2_O before start of the OLA protocol.

Heart rate was 87 ± 4 bpm and mean arterial pressur (MAP) was 87 ± 3 mmHg after baseline.

### Induction of lung injury

An average of two too three lavages were needed to reach the target of PaO_2_ < 100 mmHg at F_I_O_2_ 1.0 15 min after the last lavage.

After induction of lung injury, the PaO_2_/F_I_O_2_-ratio decreased significantly to 68 ± 10 mmHg. In conjunction, PaCO_2_ increased to 65 ± 4 mmHg at lung injury baseline. Arterial pH was 7.272 before the start of the automatized ventilatory protocol.

PIP increased to 29 ± 3 cm H_2_O, whereas measured PEEP was unchanged before start of closed-loop ventilation. Tidal volumes were 5.7 ± 0.2 ml/kg BW after injury induction. This was mirrored by a decrease in dynamic compliance to 8.3 ± 1.4 ml/cm H_2_O.

After lung injury, heart rate was 100 ± 12 bpm and MAP was 96 ± 5 mmHg before start of the OLA protocol.

### Effects of closed-loop OLA ventilation on blood gas-, respiratory- and hemodynamic variables over time

**Fig. 2:**
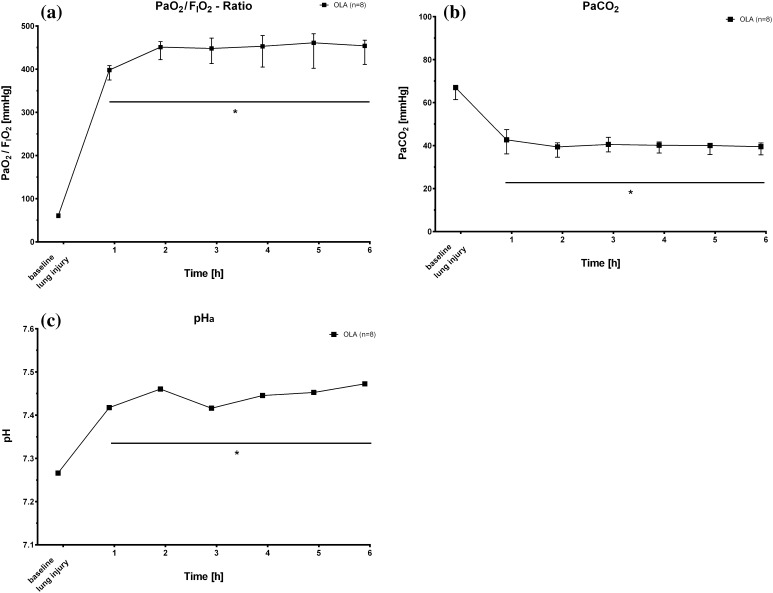
**a**–**c** Time course after induction of lung injury (baseline lung injury) of PaO_2_/F_I_O_2_-ratios, PaCO_2_ and arterial pH measurements for animals ventilated according to the open lung algorithm (OLA). F_I_O_2_, fraction of inspired oxygen; PaO_2_, arterial partial pressure of oxygen; PaCO_2_, arterial partial pressure of carbon dioxide; pH, negative decimal logarithm of H^+^ ion concentration. Values are median and interquartile range except for pH values. pH values are depicted as the negative decimal logarithm of the mean H^+^ ion concentration. *P < 0.05 versus baseline lung injury

Oxygenation of arterial blood improved significantly immediately after the start of closed-loop mechanical ventilation. After one hour of ventilation adhering strictly to the open lung approach, the PaO_2_/F_I_O_2_-ratio was 385 ± 13 mmHg. Over the time course of the experiment, the magnitude of these changes remained constant with a mean PaO_2_/F_I_O_2_-ratio range of 385–458 mmHg during OLA ventilation. Decarboxylation of arterial blood was more efficient with use of the OLA ventilatory strategy as compared to baseline ventilation after lung injury. After 1 h, arterial PCO_2_ was reduced to 43 ± 3 mmHg and PaCO_2_ was tightly controlled over time in OLA treated animals (range of means: 39–42 mmHg). In line with these data, arterial pH was 7.42 one hour after the start of automatized mechanical ventilation in the OLA group. During the following 5 h of the experiment, pHa increased to a mean of 7.484 with automatized OLA ventilation. Figure 2a–c depict these changes.

**Fig. 3:**
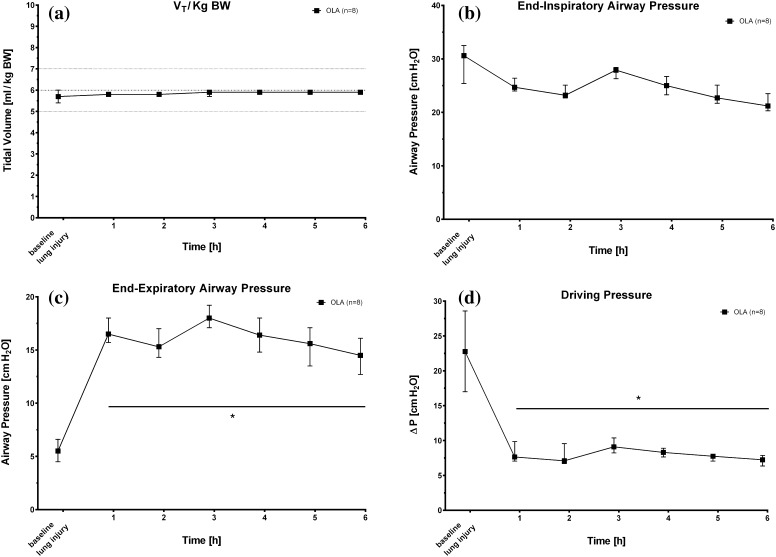
**a–c** Time course after induction of lung injury (baseline lung injury) of applied tidal volumes, end-inspiratory and end-expiratory airway pressures and lung driving pressures (ΔP) for animals ventilated according to the open lung algorithm (OLA). *V*_*T*_ tidal volume, *∆P* driving pressure. Values are median and interquartile range. *P < 0.05 versus baseline lung injury

The closed-loop mechanical ventilation protocol provided instantaneous and strict adherence to low tidal volume ventilation. Beginning immediately with the start of the experimental protocol, measured tidal volumes were 5.8 ± 0.1 ml/kg BW after one hour of closed-loop ventilation. Tidal volumes at the end of the experiment were 5.9 ± 0.1 ml/kg BW (Fig. 3a). The inspiratory pressures necessary to deliver these tidal volumes one hour after lung injury were 26 ± 1 cm H_2_O (PIP). Over the next 5 h of mechanical ventilation, PIP decreased to 22 ± 2 cm H_2_O in OLA treated pigs at the end of the experiments (Fig. 3b). The applied PEEP one hour after the start of closed-loop ventilation was 18 ± 1 cm H_2_O in pigs ventilated by the implemented OLA algorithm. The PEEP value at the end of the experiment was 15 ± 1 cm H_2_O. Figure 3c depicts the PEEP values chosen by the automatized OLA algorithm over time. The sum of the above described PIP- and PEEP values is that the applied driving pressures (∆P) were low at all times of the experiment (Fig. 3d). Despite high driving pressures at lung injury baseline (22 ± 3 cm H_2_O), the ∆P was 8 ± 1 cm H_2_O in OLA treated animals 1 h after lung injury. These low driving pressures were consistently measured until the end of the experiment with a final value of 7 ± 1 cm H_2_O with automatized ventilation following the principles of the open lung concept.

**Fig. 4:**
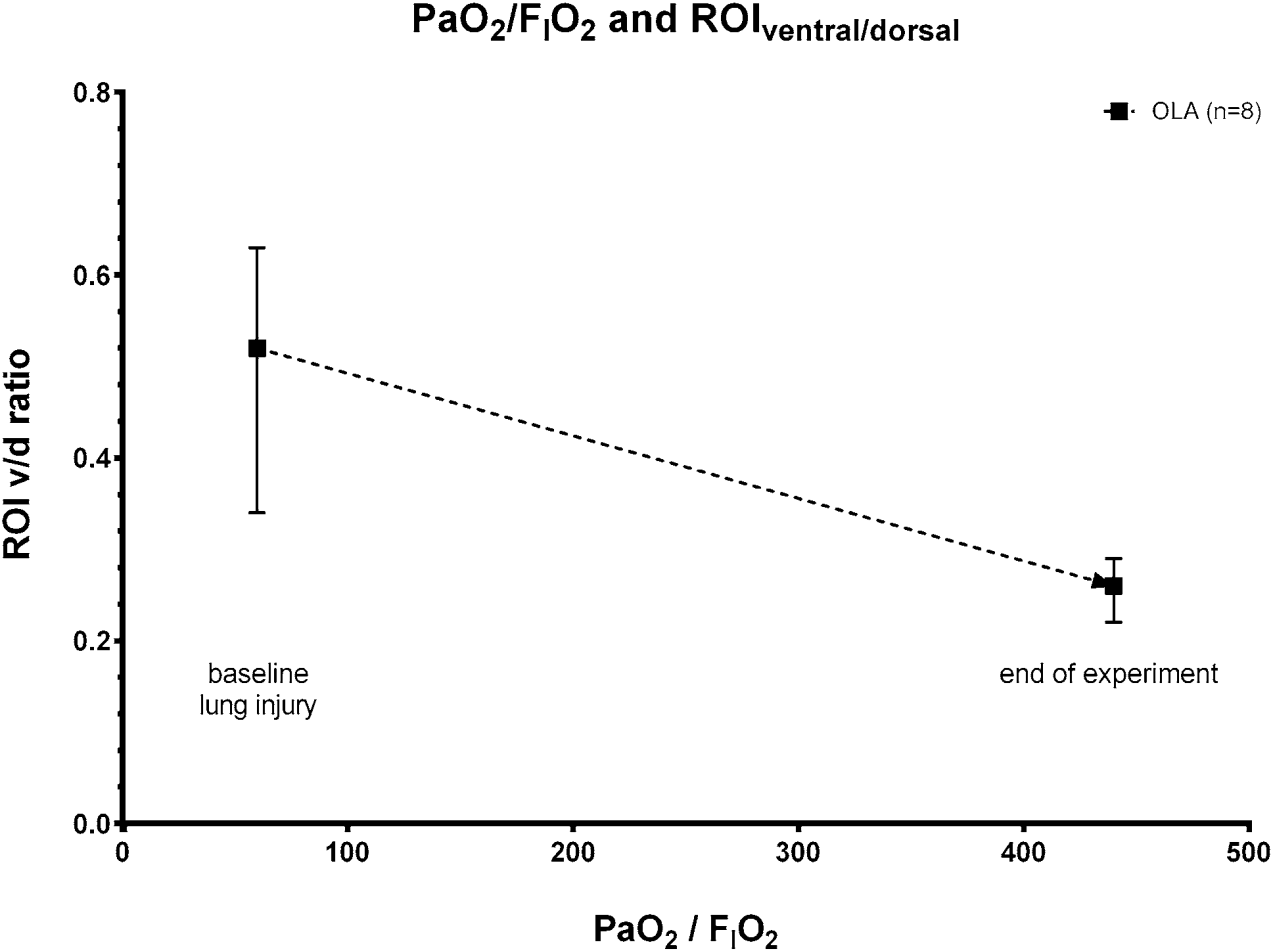
Arterial partial pressure of oxygen/fraction of inspired oxygen (PaO_2_/F_I_O_2_) and ratio of ventral/dorsal (V/D) ventilation changes in the ventral- and dorsal region of interest (ROI) after lung injury induction (baseline lung injury) and at the end of the experiment. Values are medians and interquartile ranges of animals ventilated according to the open lung algorithm (OLA). *F*_*I*_ *O*_*2*_ fraction of inspired oxygen, *PaO*_*2*_ arterial partial pressure of oxygen, *V*/*D* ratio of ventral/dorsal ventilation changes, *ROI* region of interest

Regional ventilation changes as measured by EIT in ventral (ROI I), medial (ROI II) and dorsal (ROI III) lung regions are given in table I. Time course of changes in regional ventilation and graphical illustrations of EIT measurements can be found in the supplemental digital content to this manuscript. When analyzing the ratio of ventral to dorsal ventilation over the measured PaO_2_/F_I_O_2_-ratios, we found that increased ventilation in dorsal lung regions (ROI III) mainly accounted for the improvement in blood oxygenation using the OLA protocol (Fig. 4).

Heart rate and mean arterial pressure did not change over time (data not shown).

## Discussion

Using a closed-loop feedback algorithm for mechanical ventilation, our key findings are that (a) the OLA algorithm allows for fully automated mechanical ventilation without any human intervention in severely lung injured pigs by (b) successfully providing lung protective mechanical ventilation and that (c) application of the automatized “open lung approach” results in high PaO_2_/F_I_O_2_-ratios and low driving pressures in conjunction with dorsal lung recruitment and ventilation.

To test whether a fully automatic closed-loop “open lung” ventilatory strategy is feasible and to measure the effects of such ventilation on gas exchange, respiratory- and hemodynamic parameters in conjunction with measurements of ventilation distribution (EIT), we chose to work in the lavage-induced lung injury model [10].

First, we found that automatic mechanical ventilation is feasible and securely provides lung protective ventilation without any human intervention. Several studies now show that in times of economic restriction of human resources, the physician staffing pattern significantly affects clinical outcomes of critically ill patients [5]. Of relevance, the recent LUNG SAFE study found that more than one-third of all patients with ARDS still receive tidal volumes of more than 8 ml/kg of predicted body weight (PBW), that the selected PEEP level was constantly low across the spectrum of ARDS severity and that hypoxemia was predominantly treated by increasing the F_I_O_2_ [11]. In the light of the remaining high overall mortality rate of 40% of patients with ARDS, we argue that any automatic ventilatory mode that guarantees strict control of tidal volumes in conjunction with appropriate PEEP levels, might help to reduce the incidence of volu-, baro and atelectrauma to the lung. Thus, closed-loop physiological feedback mechanical ventilation might help to prevent ventilator-induced lung injury (VILI) without imposing additional health care costs.

Second and with regards to the selection of PEEP, our data show that with application of a fully computer controlled “open lung” approach, the absolute level of positive end expiratory pressure was higher as compared to the ARDS Network PEEP/F_I_O_2_ table (see [1] and Fig. 3c) and higher as compared to our previous closed loop ARDSNet algorithm [6]. Application of higher PEEP was accompanied by a high ratio of P_a_O_2_/F_I_O_2_ and near normal arterial blood PCO_2_ and pHa values. Of importance, ventilation with the OLA protocol also reduced the difference between end-expiratory and end-inspiratory airway pressure (see Fig. 3b, c). While better oxygenation and decarboxylation are consistently reported in animal work of higher PEEP during mechanical ventilation for ARDS therapy, the application of higher PEEP levels in conjunction with recruitment maneuvers in the LOVs study did not reduce all-cause mortality in patients with ARDS [12]. Instead, only a secondary analysis of these data showed that a positive oxygenation response to positive end-expiratory pressure predicted mortality in this patient cohort [3]. To this end, Amato et al. recently analyzed data from 3562 patients enrolled in nine randomized trials of mechanical ventilation for ARDS treatment, and found that the risk of in-hospital death was reduced only when the chosen PEEP level resulted in a smaller driving pressure (∆P = V_T_/Crs) of the lung [13]. This work clearly showed that scaling the tidal volume to the actual compliance of the respiratory system (the so called “baby lung concept”) [14] is a better predictor of lung protective ventilation, than scaling the tidal volume to the predicted body weight of the patient. These findings were again supported by Bellani et al. showing increased mortality with increasing quintiles of driving pressure in ARDS patients [11]. Although our implemented “open lung” algorithm did not aim for the lowest possible driving pressure, these findings are important to our data. We found that automatic application of higher PEEP in conjunction with a lung recruitment strategy resulted in low peak inspiratory airway pressures. Thus, the closed-loop “lung open” approach lead to very low driving pressures without reduction of tidal volumes beneath the values proposed by the ARDS-Network investigators. In other words, the tidal volume in the “lung open” protocol was better scaled to the actual compliance of the respiratory system. Moreover, our data from the EIT measurements show increased ventilation to dorsal lung areas accompanied by slightly smaller tidal volumes to more ventral lung regions (Table 1; Fig. 3d). Thus, we explain the reduction of lung driving pressures by increased recruitment of the dorsal lung, leading to a larger functional lung size for any given tidal volume.

**Table 1:** Regional changes of ventilation in ventral, medial and dorsal lung regions

		ROI I (%)	ROI II (%)	ROI III (%)
Lung injury baseline	OLA	13 (12–15)	62 (56–63)	25 (23–34)
End of experiment	OLA	11 (10–11)	49 (46–50)	41 (39–42)

Median (interquartile range) regional ventilation using closed loop open lung approach (OLA) ventilation in ventral (ROI I), medial (ROI II) and dorsal (ROI III) lungs regions after lung injury and at the end of the experiment

### Study limitations

Our study has several important limitations that need to be addressed:

First, the used lavage-induced lung injury model is based on the depletion of surfactant from the alveolar surface area [15]. Thus, the severe gas exchange impairment and reduction of respiratory system compliance are caused mainly by atelectasis of dorsal (basal) lung regions. These lung areas have a high potential to be recruited by ventilatory maneuvers that include short-term application of high inspiratory pressures to open up the lung, followed by application of higher than previous PEEP levels to keep the lung open [4]. Maintaining the lung open is important not only for oxygenation targets, but also for avoidance of cyclic opening and closing of already damage alveoli (shear force reduction), surfactant activity preservation and lung edema reduction. Yet, the recruitability of the severely injured lung in the intensive care unit is usually much lower than in this artificial animal model. Our findings are therefore not generalizable to all patients suffering from ARDS [16]. In addition, other rescue maneuvers for ARDS therapy may be less invasive and may be equally beneficial as the “brut force” approach of recruitment maneuvers by high inflation pressures. For example, prone positioning shifts the “baby lung” in ARDS from ventral to dorsal lung regions, leading to more homogenously distribution of trans-pulmonary forces, reduction of “true shunt” (oxygenation improvement) and ultimately to survival benefit [17, 18]. Yet, recruitment maneuvers are more often used as a rescue therapy than prone positioning during ARDS therapy (recruitment: 20.9% vs. proning: 7.9% in the LUNG SAFE study) [11] and we believe that standardization and automation of recruitment maneuvers may help to clarify their role in the treatment of ARDS patients. To this end, we would like to point out that—while no known safe upper limit for the applied peak recruitment pressures exists—we advise against direct translation of our findings to humans and against the routine use of very high peak pressures (up to 60–70 cm H_2_O as described in our protocol). Only a careful and critical assessment of the risks associated with peak inflation pressures (baro- and volutrauma) for each individual patient will help to prevent the possible injurious and detrimental side effects associated with high transpulmonary pressures to recruit the injured lung.

Second, the time scale of our experiments did not allow us to identify the optimal setting for increases, maintenance and reduction of PEEP in the automated protocol. Of note, our fast responding intra-arterial saturation measurements allowed us to increase both PIP and PEEP whenever we diverted from the pre-specified oxygenation target. In our case, the computer increased both PIP and PEEP in 30 s intervals until the target value for SpO_2_ was reached. These fast increases may have deleterious consequences for pulmonary and systemic arterial blood pressure due to the complex interaction of mechanical ventilation, right ventricular function and blood flow to systemic organs [19]. In addition, while the ARDS Network study does not provide clear rules for the time spent on any given F_I_O_2_/PEEP combination, fast reductions of PEEP will lead to derecruitment and subsequent loss of aerated lung tissue, increased ventilation/perfusion (V/Q) mismatch and ultimately hypoxemia. Importantly, while ensuring sufficient oxygenation, the selected F_I_O_2_ from “table rules” may obscure clinically relevant cyclic opening and closing of alveoli and thus contribute to the development of VILI. Any automation of mechanical ventilation needs to be carefully investigated in the light of these important biologic consequences.

Third, our study was performed in deeply anesthetized and paralyzed pigs. This does not reflect current practice of ICU management of mechanical ventilation and is against accumulating evidence for reduction of sedation to improve survival and prevention of ICU-acquired muscle weakness in the critically ill. As to the use of neuromuscular blockers, Papazian et al. showed that early administration of a neuromuscular blocking agent improved the adjusted 90-day survival rate and increased the time off the ventilator [20]. Reduction of muscle oxygen consumption and cardiac output, an increase in functional residual capacity as well as a decreased production of pro-inflammatory cytokines are the classical explanation for this beneficial effect. The most reasonable cause of effect yet seems to be the prevention patient-ventilator dyssynchrony, reduction of high transpulmonary pressures during spontaneous breathing and prevention of Pendelluft effects from apical to dorsal lung regions [21, 22]. Since we have no data on the effects of spontaneous breathing during closed-loop feedback control of mechanical ventilation, we can only comment that future modes of automatized mechanical ventilation will need to allow for spontaneous breathing while still providing adherence to a low tidal volume- and low trans-pulmonary pressure ventilatory strategy.

Fourth, we did not directly compare the OLA protocol to a closed loop protocol based on the ventilatory settings of the ARDS-Network landmark study. Yet, when comparing the recent findings to a previously published (historic) closed loop ARDSNet algorithm, we find that the open lung protocol leads to higher PaO_2_/F_I_O_2_-ratios and significant reduction of lung driving pressures via dorsal recruitment and ventilation of the lung [6].

## Conclusions

In conclusion, we found that automatic closed-loop mechanical ventilation following the “open lung” approach is feasible in severe lavage-induced lung injury in pigs. This “open lung” ventilation leads to high PaO_2_/F_I_O_2_-ratios and significant reduction of lung driving pressures via dorsal recruitment and ventilation of the lung.
